# Integration of Structural Variant Data into the NIAGADS Alzheimer's Genomics Database: Enhancing Understanding of Alzheimer's Disease Mechanisms

**DOI:** 10.1002/alz70855_103672

**Published:** 2025-12-24

**Authors:** Emily Greenfest‐Allen, Sam Tate, Hui Wang, Po‐Liang Cheng, Zivadin Katanic, Otto Valladares, Wan‐Ping Lee, Li‐San Wang

**Affiliations:** ^1^ Penn Neurodegeneration Genomics Center, Department of Pathology and Laboratory Medicine, Perelman School of Medicine, University of Pennsylvania, Philadelphia, PA, USA

## Abstract

**Background:**

Structural variants (SVs) are large genomic alterations that can profoundly impact disease risk by disrupting gene function and regulation. Despite this, their role in Alzheimer's disease (AD) remains understudied due to challenges to accurate detection and prediction of potential impact. A recent Alzheimer's Disease Sequencing Project (ADSP) study addressed many of these challenges and identified >400k SVs (168,223 high quality) from the sequencing of ∼16k whole genomes. The study detected a burden of singletons and homozygous deletions in AD cases, as well as an association of protein‐altering SVs with several known AD genes (H. Wang et al.; doi:10.1101/2023.09.13.23295505). This work highlights the essential role of SVs in AD genetics, emphasizing that further identification and analysis are needed. To support this, we have integrated these results into the NIA Genetics of Alzheimer's Disease Data Storage Site's (NIAGADS) Alzheimer's Genomics Database (GenomicsDB).

**Method:**

Part of NIAGADS's Open Access Data Initiative, the GenomicsDB compiles unrestricted AD‐relevant genetic data and annotations, making them more accessible to AD‐researchers and facilitating data reuse. SVs detected in the ADSP study were harmonized with existing GenomicsDB data and each was assigned a unique identifier based on genomic location and variant type. The dataset was then augmented by detecting overlaps with SVs reported in key third‐party databases (e.g., dbVar, gnomAD). Significant associations with known AD‐genes detected in the analysis were flagged, and all variants were annotated using VEP and annotSV. Protein‐altering variants were identified based on predicted consequences, and the relative functional impact of the SVs ranked.

**Result:**

In its next release, the GenomicsDB will make public SV reports that compile both annotations and information on other genomic features and AD‐genetic associations overlapping the variant span. An annotated SV genome browser track was also generated.

**Conclusion:**

Identification and analysis of SVs in AD is essential for gaining a more comprehensive understanding of the genetic underpinnings of this complex disease. By making these data accessible and placing them in the broader genomic context, inclusion of the ADSP Structural Variant study results in the GenomicsDB creates a unique and valuable resource for AD‐researchers.

